# Land subsidence analysis along high-speed railway based on EEMD-Prophet method

**DOI:** 10.1038/s41598-024-51174-9

**Published:** 2024-01-06

**Authors:** Qiu Dongwei, Tong Yuci, Wang Yuzheng, Ding Keliang, Liu Tiancheng, Wan Shanshan

**Affiliations:** 1 School of Geomatics and Urban Spatial Informatics, Beijing University of Civil Engineering and Architecture, Beijing, 100044 China; 2 School of Electrical and Information Engineering, Beijing University of Civil Engineering and Architecture, Beijing, 100044 China

**Keywords:** Civil engineering, Scientific data, Environmental impact

## Abstract

Environmental changes and ground subsidence along railway lines are serious concerns during high-speed railway operations. It is worth noting that AutoRegressive Integrated Moving Average (ARMA), Long Short-Term Memory (LSTM), and other prediction methods may present limitations when applied to predict InSAR time series results. To address this issue, this study proposes a prediction method that decomposes the nonlinear settlement time series of feature points obtained through InSAR technology using Ensemble Empirical Mode Decomposition (EEMD). Subsequently, multiple Intrinsic Mode Functions (IMFs) are generated, and each IMF is individually predicted using the Prophet forecasting model. Finally, we employ an equal-weight superimposition method to combine the results, resulting in the prediction of the InSAR settlement time series. The predicted values of each component are subsequently weighted equally and combined to derive the final prediction outcome. This paper selects InSAR monitoring data along a high-speed railway in inland China and uses the proposed method and ARMA and Prophet models to carry out comparative experiments. The experimental results show that compared with the ARMA and Prophet models, the method in this paper improves the root mean square error by 58.01% and 32.3%, and increases the mean absolute error by 62.69% and 33.78%, respectively. The predicted settlement values generated by our method exhibit better agreement with the actual InSAR monitoring values.

## Introduction

High-speed rail plays an important role in connecting cities for socio-economic development. Subsidence in the area along the high-speed railway will have a certain negative impact on the stability and safety of the railway, and then affect the normal operation of the railway^1^. Therefore, to ensure the safety of train operations, it is essential to monitor changes in infrastructure such as high-speed railways and predict their changing trends^2^.

In recent years, many experts and scholars have researched on surface subsidence prediction and achieved meaningful results. Aiming at the problem that improper selection of training samples will directly affect prediction accuracy, Zhou Dingyi^3^ constructed a land subsidence prediction method based on a neural network algorithm from the perspective of multiple factors. Zhou Qihang^4^ proposed an improved third-order gray prediction model combined with terrain factors and neural network error correction for urban land subsidence prediction. Using a neural network to predict land subsidence will achieve higher precision. However, in the prediction process, there will be problems such as an unstable prediction process, complex parameter setting selection, and only suitable for use in a single environment. Many classic traditional forecasting models, such as ARMA^5^and ARIMA^6^, can also be employed to predict future time series changes. However, the predictive accuracy of these models is often unsatisfactory, and the accuracy needs to be maintained through a rolling forecast. Yang Zhengrong^7^ used the glacier deformation field and gray correlation analysis obtained by InSAR technology and revealed the relationship between glacier deformation and various factors such as temperature, precipitation, and slope. Xiong Zhiqiang^8^ used the statistical properties of curve fitting to select the best curve model from several candidate curve models to predict the subsidence time series. Shi Xianlin^9^ used the regenerative settlement information combined with InSAR technology and Terzaghi theory and the corresponding geological parameters of the artificial airport to successfully deduce the predicted settlement curve of the offshore artificial island airport. Chen Yi^10^ used the LSTM neural network to successfully establish a time series InSAR prediction model when analyzing the Beijing Capital Airport using the InSAR monitoring results. Since spatial heterogeneity can interfere with the accuracy of large-scale land subsidence prediction, Liu Qinghao^11^ proposed the HLSTM method that accounts for spatial heterogeneity, thereby mitigating the influence of spatial heterogeneity on the prediction results of large-scale land subsidence. However, using the above method combined with InSAR to predict the subsidence field needs to establish a complex model and consider the influence of various factors. If some factors are not taken into consideration, it may cause significant errors in the prediction results and other shortcomings^12^.

The Prophet model^13^ is a time series forecasting model based on an additive model. In 2017, Taylor et al. proposed a new signal-processing method and released an open-source software package. This model employs a generalized additive model to conduct Bayesian curve fitting of nonlinear trends on time series data, thus offering remarkable flexibility and robustness^14^. The EEMD^15^ is based on the empirical mode decomposition^16^ (EMD) method, which effectively addresses the modal aliasing of the IMF obtained via EMD decomposition by superimposing Gaussian white noise^17^. The core concept is to perform multiple decompositions on different scales of the input data. By averaging the outcomes of each decomposition, the randomness and problems with local extreme values in EMD decomposition are reduced, and the decomposition results are more stable.

Aiming to address the aforementioned issues, this study proposes a prediction method that decomposes InSAR nonlinear time series features before predicting them. Specifically, the settlement time series obtained by InSAR is firstly decomposed into intrinsic mode functions using the EEMD method, which can characterize local features at different times. Then, the Prophet model is used to predict each intrinsic mode function. Finally, the predicted results of each intrinsic mode function are combined with equal weights to enhance the prediction accuracy.

## Method

### Main idea

**Figure 1:**
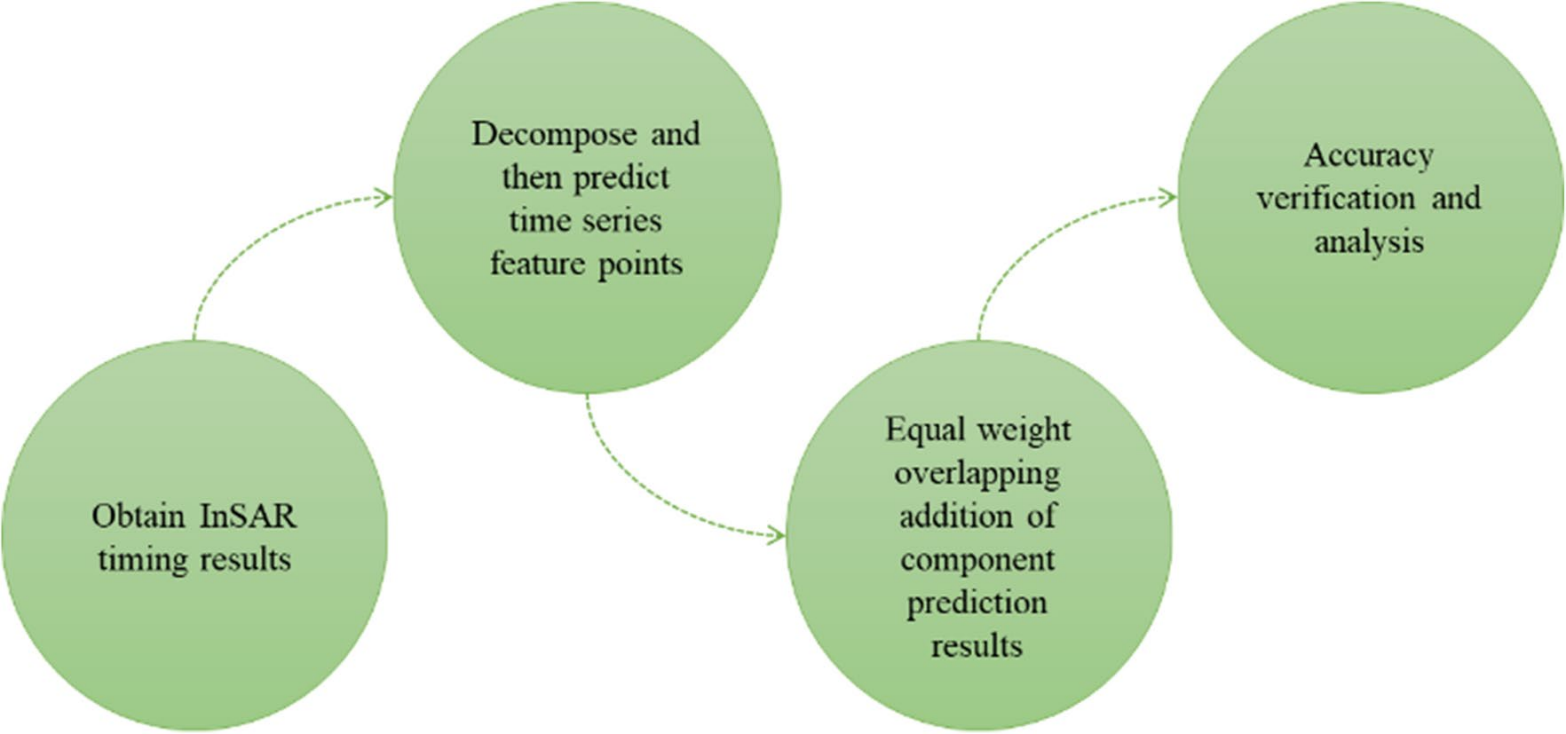
Technical flow chart.

The method proposed in this paper is based on the SBAS-InSAR technology to predict the settlement time series characteristics of InSAR settlement. SBAS-InSAR was originally proposed by Berardino et al.^18^. This technology optimizes the combination of images acquired at different times, and its main process is shown in the literature^19^. In our approach, we first perform EEMD decomposition of the SBAS-InSAR time series results. Subsequently, we make separate predictions for each of the decomposition results, and finally, we apply a novel method involving equal-weight superposition. Our method combines the strong adaptability, fast prediction speed, and high prediction effect of the Prophet model with the EEMD method's characteristics of not requiring prior conditions. Figure 1 illustrates the flow chart of our method for predicting InSAR nonlinear subsidence time-series data, and the main steps are as follows:

1. Conduct SBAS -InSAR processing on the study area to obtain the InSAR subsidence time series results.
2. Perform EEMD decomposition on the nonlinear time series results to obtain IMF components, and use the Prophet model to predict each IMF component separately.
3. Superimpose the prediction results of each IMF with equal weight to obtain the prediction results of the settlement point.
4. Evaluate and analyze the prediction accuracy of the settlement point.

### SBAS-InSAR Processing Workflow

SBAS-InSAR is a monitoring technology based on traditional D-InSAR technology, which is further deepened to obtain the time series characteristics of surface deformation. The algorithm selects N + 1 SAR images, sets an appropriate spatiotemporal baseline consistent with PS-InSAR, generates N small baseline set differential interference pairs, and performs phase unwrapping after generating differential interference patterns.

During this process, we set Multilook Number to 4 where Range Looks is 4 and Azimuth Looks is 1. This reduces the impact of coherence spots and achieves a good balance between signal-to-noise ratio and spatial resolution. In addition, in order to eliminate atmospheric noise, Goldstein filtering is used, and its window size is set to 5*5. The selection of Unwrapping Method Type is Minimum Cost Flow, where Unwrapping Decomposition Level is 1 and Unwrapping Coherence Threshold is 3. These parameter choices were guided by careful consideration of the trade-offs between computational efficiency and preserving deformation signals in the presence of atmospheric effects.

It is assumed that within the ordered time $\left( {t_{1} ,t_{2} ,...t_{n} } \right)$ of the interest area, N + 1 SAR images of the same orbit are acquired, and the auxiliary images are registered to the common main image according to the interference conditions, and then the auxiliary images are registered to the common main image according to the interference conditions. The complex phase solution is performed on the image, and M differential interference patterns are finally generated. Among them, M needs to meet the following conditions:

1 $$\frac{{\left( {{\text{N}} + 1} \right)}}{2} \le {\text{M}} \le {\text{N}}\left( {\frac{{{\text{N}} + 1}}{2}} \right)$$

During the interference process, in the jth differential interference pattern generated by the two SAR images acquired at $t_{1}$ and $t_{2}$ ($t_{1}$ > $t_{2}$) as the main and auxiliary images, the interference phase of any pixel can be expressed as:

2 $$\begin{array}{*{20}c} {\delta \phi_{j} \left( {x,r} \right) = \phi \left( {t_{1} ,x,r} \right) - \phi \left( {t_{2} ,x,r} \right)} \\ { \approx \frac{4\pi }{\lambda }\left[ {d\left( {t_{1} ,x,r} \right) - d\left( {t_{2} ,x,r} \right)} \right] + \Delta \phi_{{j_{{top{ }}} }} + \Delta \phi_{{j_{atm} }} + \Delta \phi_{{noise{ }}} } \\ \end{array}$$

In the formula, $\lambda$ is the wavelength, $d\left( {t_{1} ,x,r} \right)$ and $d\left( {t_{2} ,x,r} \right)$ are the cumulative deformation quantities, $\Delta \phi_{{j_{{top{ }}} }}$ is due to the reference DEM Terrain phase error caused by inaccuracy, $\Delta \phi_{{j_{atm} }}$ is the error phase caused by atmospheric delay, $\Delta \phi_{{noise{ }}}$ is the error phase caused by the influence of noise. After removing the terrain phase error, the error caused by atmospheric delay, and the error caused by the influence of noise, through simplified formula (3), the interference phase can be expressed as:

3 $$\delta \phi_{j} \left( {x,r} \right) = \phi \left( {t_{1} ,x,r} \right) - \phi \left( {t_{2} ,x,r} \right) \approx \frac{4\pi }{\lambda }\left[ {d\left( {t_{1} ,x,r} \right) - d\left( {t_{2} ,x,r} \right)} \right]$$

To obtain the sedimentation sequence, the average phase velocity can be expressed as:

4 $${\text{V}}_{{\text{j}}} = \frac{{\upphi_{{\text{j}}} - \upphi_{{{\text{j}} - 1}} }}{{{\text{t}}_{1} - {\text{t}}_{{{\text{j}} - 1}} }}$$

In the formula, the phase value of the jth interference pattern can be expressed as:

5 $$\mathop \sum \limits_{{k = t_{B} ,j = 1}}^{{t_{A} ,j}} \left( {t_{k} - t_{k - 1} } \right)v_{k} = \delta \phi$$

The deformation of the time series calculated by the SBAS-InSAR algorithm can be expressed as:

6 $$A_{v} = \delta \phi$$

**Figure 2:**
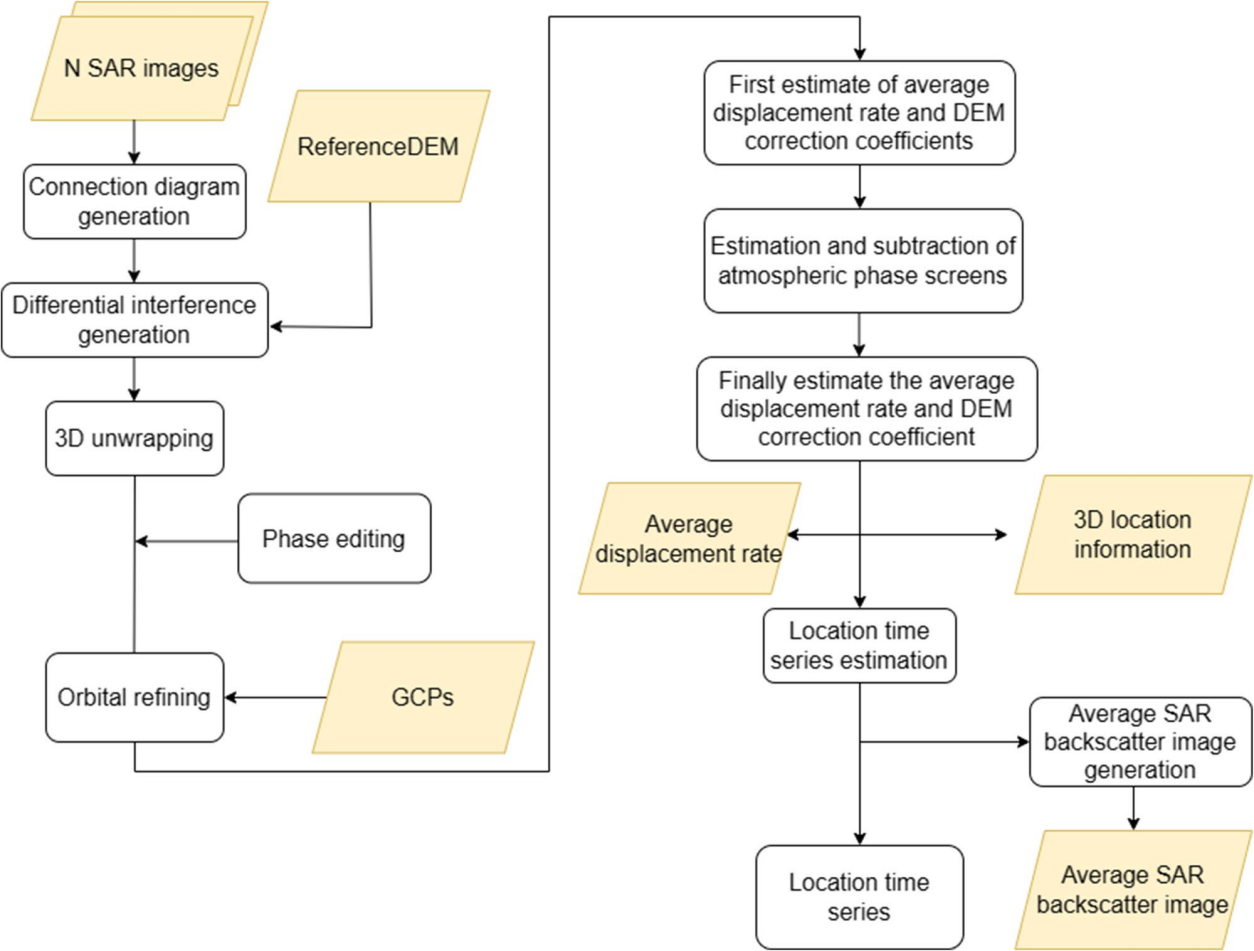
SBAS technical processing flow.

In the formula, A is the MXN matrix, and 80 is the vector representing the interference phase value. The SBAS-InSAR technical data processing flow chart is shown in Fig. 2.

### Prophet prediction model based on EEMD

The Prophet prediction method, based on EEMD, combines the advantages of both techniques. It can better capture the nonlinear, non-constant, and periodic changing characteristics of the time series while effectively removing noise and interference through decomposition. This approach enhances prediction accuracy while preserving the important information of the original signal. In line with the proposed EEMD-Prophet prediction method, the original signal of the InSAR monitoring point is firstly decomposed into several IMF and residual components using the EEMD method, specifically:

(1) Add a set of Gaussian white noise n(t) to the InSAR settlement time series signal feature Q(t) to obtain an overall signal X(t)

7 $${\text{X}}\left( {\text{t}} \right) = {\text{Q}}\left( {\text{t}} \right) + {\text{n}}\left( {\text{t}} \right)$$

(2) Decompose X(t) by EMD first to obtain the corresponding IMF of each order, and its formula is expressed as:

8 $${\text{X}}\left( {\text{t}} \right) = \mathop \sum \limits_{{{\text{i}} = 1}}^{{\text{n}}} {\text{IMF}}_{{\text{i}}} \left( {\text{t}} \right) + {\text{res}}_{{\text{i}}} \left( {\text{t}} \right)$$

(3) Add a set of different white noise signals $n_{j} \left( {\text{t}} \right)$ to the InSAR settlement time series feature ${\text{Q}}\left( {\text{t}} \right)$ to obtain another overall signal $X_{j} \left( {\text{t}} \right)$. After performing EMD decomposition on $X_{j} \left( {\text{t}} \right)$, obtain the corresponding IMF components of each order using its formula expression:

9 $${\text{X}}_{{\text{j}}} \left( {\text{t}} \right) = \mathop \sum \limits_{{{\text{i}} = 1}}^{{\text{n}}} {\text{IMF}}_{{{\text{ji}}}} \left( {\text{t}} \right) + {\text{res}}_{{\text{j}}} \left( {\text{t}} \right)$$

(4) Repeat the above steps, and calculate the mean value of each IMF obtained as the IMF of the final signal, and its formula is expressed as:

10 $${\text{IMF}}_{{\text{j}}} \left( {\text{t}} \right) = \frac{1}{{\text{N}}}\mathop \sum \limits_{{{\text{i}} = 1}}^{{\text{n}}} {\text{IMF}}_{{{\text{ji}}}} \left( {\text{t}} \right)$$

where ${\text{IMF}}_{{\text{i}}} \left( {\text{t}} \right)$ denotes the IMF for each specific order, whereas ${\text{res}}_{{\text{i}}} \left( {\text{t}} \right)$ corresponds to the associated residual component. N signifies the total count of iterations involving white noise addition. t is the time for obtaining InSAR results, and i, j are the number of IMFs obtained by decomposition.

According to the principle of the Prophet model, each IMF obtained by decomposing the characteristics of the InSAR subsidence timing signal by the EEMD method is adaptively decomposed into:

11 $${\text{y}}_{imf} \left( {\text{t}} \right) = {\text{g}}_{imf} \left( {\text{t}} \right) + {\text{s}}_{imf} \left( {\text{t}} \right) + {\text{h}}_{imf} \left( {\text{t}} \right) + {\upvarepsilon }_{{\text{t}}}$$

In Eq. (11), the Prophet model decomposes each IMF signal time series feature ${\text{y}}_{imf} \left( {\text{t}} \right)$ into individual elements: the time series data trend item ${\text{g}}_{imf} \left( {\text{t}} \right)$, the oscillation period item ${\text{s}}_{imf} \left( {\text{t}} \right)$, the holiday mutation term ${\text{h}}_{imf} \left( {\text{t}} \right)$, and the residual term ${\upvarepsilon }_{{\text{t}}}$. The decomposed trend item ${\text{g}}_{imf} \left( {\text{t}} \right)$ represents the changing function of the nonlinear growth component within the time series. When analyzing land subsidence time series, the trend item is commonly captured using a logistic regression function^20^. which can be represented as:

12 $${\text{g}}_{imf} \left( {\text{t}} \right) = \frac{{\text{m}}}{{1 + {\text{e}}^{{\left[ {{\text{ - k}}\left( {\text{t - l}} \right)} \right]}} }}$$

In Eq. (12), k represents the settlement growth rate, l denotes the displacement, and m represents the upper limit of the settlement trend. As time t increases, ${\text{g}}_{imf} \left( {\text{t}} \right)$ gradually converges towards m. However, the time series of surface subsidence is subject to changes, requiring consideration of the dynamic subsidence growth rate k and the adaptive offset. Additionally, the changing growth rate needs to be taken into account. Hence, Eq. (12) is revised as:

13 $${\text{g}}_{imf} \left( {\text{t}} \right) = \frac{{\text{m}}}{{1 + {\text{e}}^{{ - \left( {{\text{k}} + {\upalpha }\left( {\text{t}} \right)^{{\text{T}}} {\updelta }} \right)\left[ {{\text{t}} - \left( {{\text{l}} + {\upalpha }\left( {\text{t}} \right)^{{\text{T}}} {\upgamma }} \right)} \right]}} }}$$

This Eq. (13) describes the variation of the trend component ${\text{g}}_{imf} \left( {\text{t}} \right)$ in the ground subsidence time series with respect to time t. In this context, the growth rate k and the offset l are dynamically changing with time, influenced by the parameters α(t), δ, and γ. This dynamic modeling allows the trend to adapt over time to better fit the observed data. The symbol t stands for time, signifying the time point. The symbol α(t) is a function related to time t. This function represents parameters that vary over time. The symbol δ is a parameter vector, signifying the variation in the growth rate k over time. The symbol γ is a parameter vector, representing the variation in the displacement l over time.

The oscillating period item ${\text{s}}_{imf} \left( {\text{t}} \right)$ includes seasonal periodic oscillations and small trend changes, and the Fourier series construction of the settlement time series data can obtain ${\text{s}}_{imf} \left( {\text{t}} \right)$, the structural form can be expressed as:

14 $$s_{imf} \left( t \right) = \mathop \sum \limits_{n = 1}^{N} \left( {a_{n} \cos \left( {\frac{2\pi nt}{T}} \right) + b_{n} \sin \left( {\frac{2\pi nt}{T}} \right)} \right)$$

**Figure 3:**
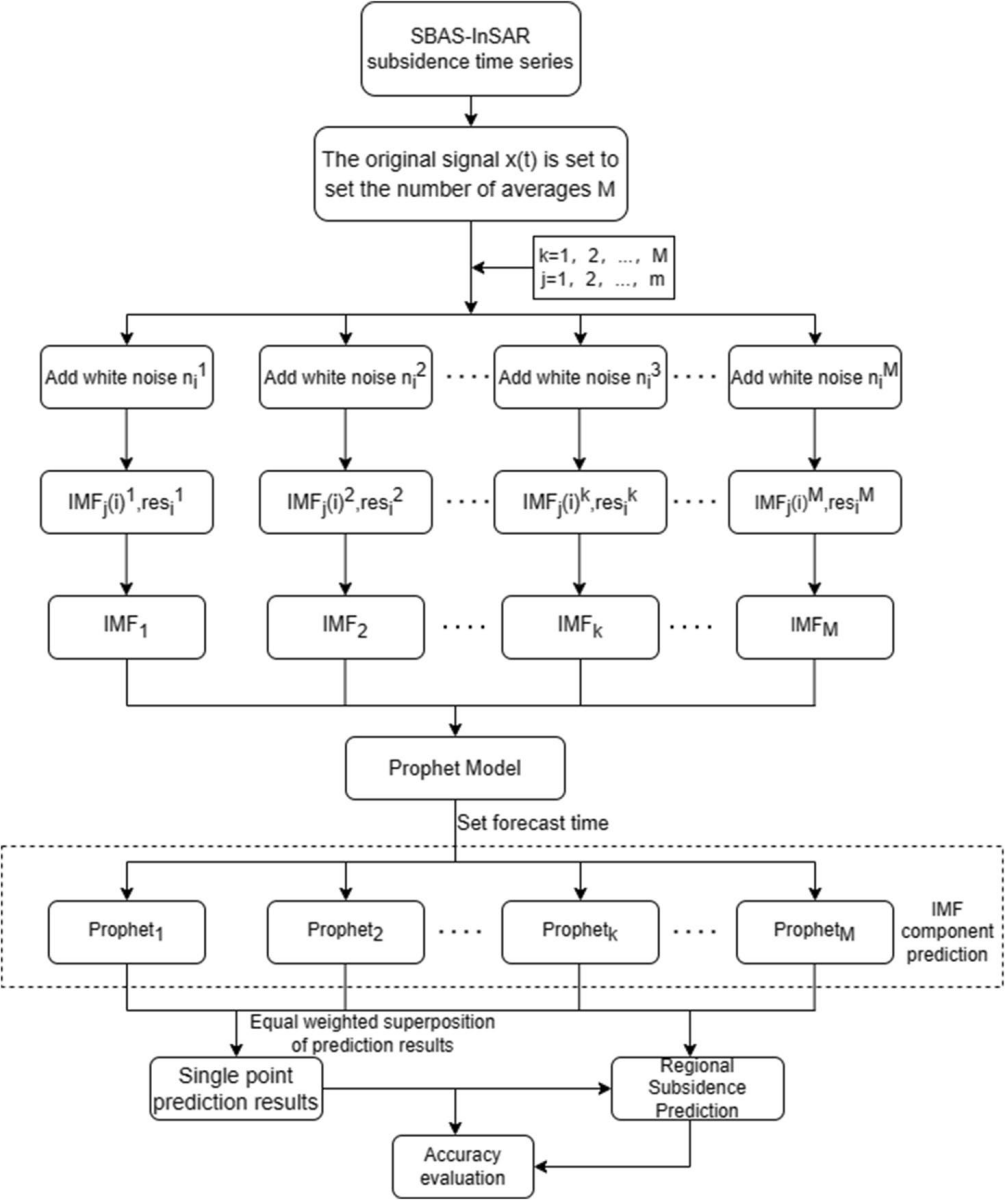
Technology Roadmap.

In Eq. (14), T represents the length of the timing cycle, while 2n denotes the anticipated number of cycles in the model. Typically, the land subsidence time series does not exhibit a holiday mutation term ${\text{h}}_{imf} \left( {\text{t}} \right)$, and thus, its influence is disregarded. The residual term ${\upvarepsilon }_{{\text{t}}}$ adheres to a normal distribution and can be interpreted as unexpected random noise and varying trends. The technical approach of the EEMD-based Prophet prediction method is illustrated in Fig. 3.

## Study areas and deformation characteristics

### Study areas and data

**Figure 4:**
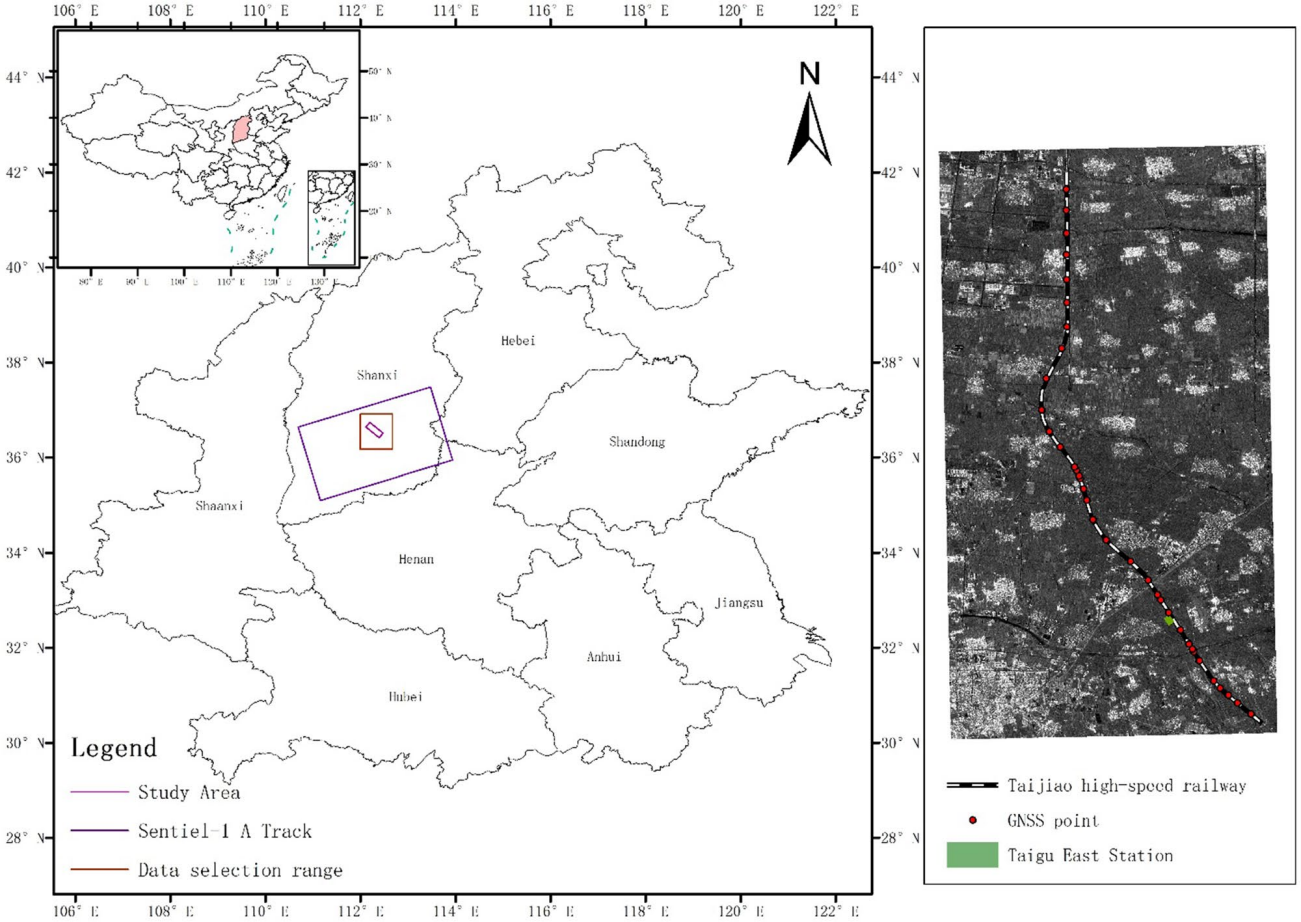
Taijiao high-speed railway research section.

The Taiyuan-Jiaozuo high-speed railway is situated in the inland region of eastern mainland China. It serves as a vital transportation link connecting Jiaozuo City in Henan Province to Taiyuan City in Shanxi Province. This railway line commenced operations on December 12, 2020, spanning a total length of 358.764 km and encompassing 13 intermediate stations. Designed for a maximum speed of 250 km per hour, the route traverses a diverse terrain comprising plains, hills, mountains, rivers, and other intricate landscapes, each presenting unique geological characteristics^21^. Notably, the railway passes through certain coal mine goaf areas, introducing additional uncertainties to train operation safety. Given the stringent requirements for track stability in high-speed rail systems, even minor surface deformations pose substantial risks to the overall operation. Figure 4 illustrates the geographical location of the research area, specifically focusing on the section of the study section spanning from Jinzhong Station to Taigu East Station along the Taijiao high-speed railway.

The data utilized in this study comprises ESA Sentinel-1A data, obtained during the satellite image period spanning from January 09, 2021, to October 07, 2022, encompassing a total of 40 scene data. The corresponding timestamps for each SAR image are presented in Table 1. The dataset is based on C-band observations with an ascending orbit direction and an incident angle of 39.27°. The imaging mode employed is the interferometric wide mode IW, with VV polarization. The pixel resolution is 5m in the range direction and 20m in the azimuth direction. The Global Navigation Satellite System (GNSS) monitoring stations are primarily deployed along the section from Jinzhong Station to Taigu East Station, as illustrated in Fig. 6, which depicts the spatial distribution of 35 observation stations. Monitoring activities commenced in July 2021.

**Table 1:** The acquisition time of SAR image data from 2021 to 2022.

Year	SAR image acquisition time											
	Jan.	Feb.	Mar.	Apr.	May	Jun.	Jul.	Aug.	Sept.	Oct.	Nov.	Dec.
2021	9	14	10	15	21	14	8	1	6	12	5	11
	21	26	22	27				13	30	24	29	23
								25				
2022	4	9	5	10	4	9	3	8	1	7		
	28	21	17	22	28	21	27		13			

### Regional deformation feature analysis

**Figure 5:**
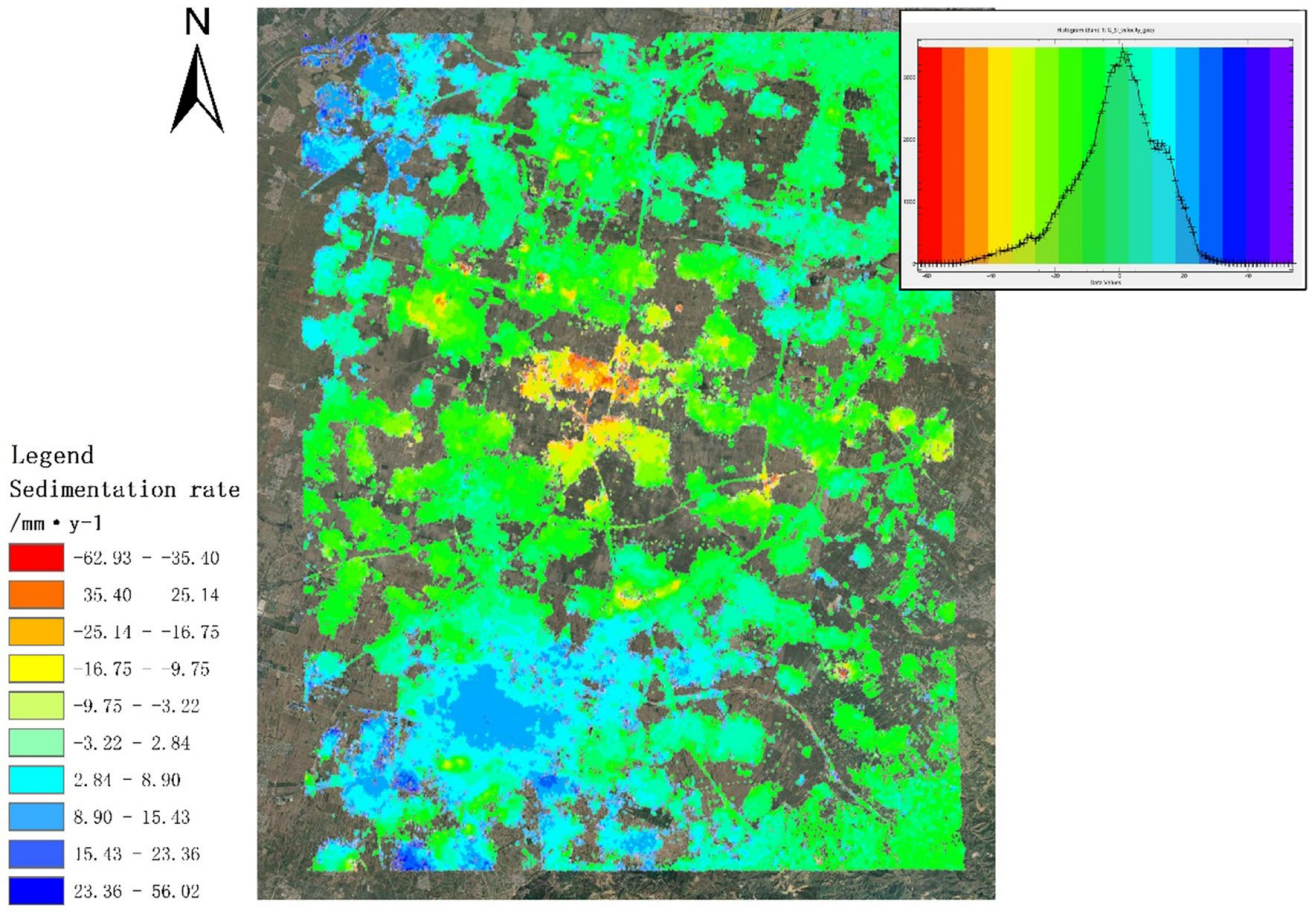
Deformation rate and histogram of the study area (the color red indicates subsidence, the color blue indicates an uplift and The color green represents relatively stable regions).

In the experiment, the SAR image captured on November 29, 2021, was selected as the primary reference image. Ground Control Points (GCP) generated by PS-InSAR were utilized for orbit refinement and re-flattening, thereby improving the accuracy of SBAS-InSAR processing^22^. Figure 5 illustrates the deformation rate results obtained by processing the research area using SBAS-InSAR and overlaying them on Google Earth Pro (V7.3.6.9345; software available at https://www.google.com/earth/about/versions/#download-pro). The histogram's peak in the upper right corner indicates that a majority of the area experiences no deformation, which aligns with the actual conditions of the selected area, providing evidence for the reliability of the SBAS-InSAR monitoring results utilizing GCP points generated by PS-InSAR. The most significant subsidence area is located at the intersection of the two railways, exhibiting an average subsidence rate of -38 mm/year. In contrast, the main uplifted areas are primarily concentrated in the southwest region, specifically the Taigucheng District, and the villages in the northwest region, particularly in the south of Liujiabao Township. These uplifted areas have average subsidence rates of 15 mm/year and 20 mm/year, respectively.

**Figure 6:**
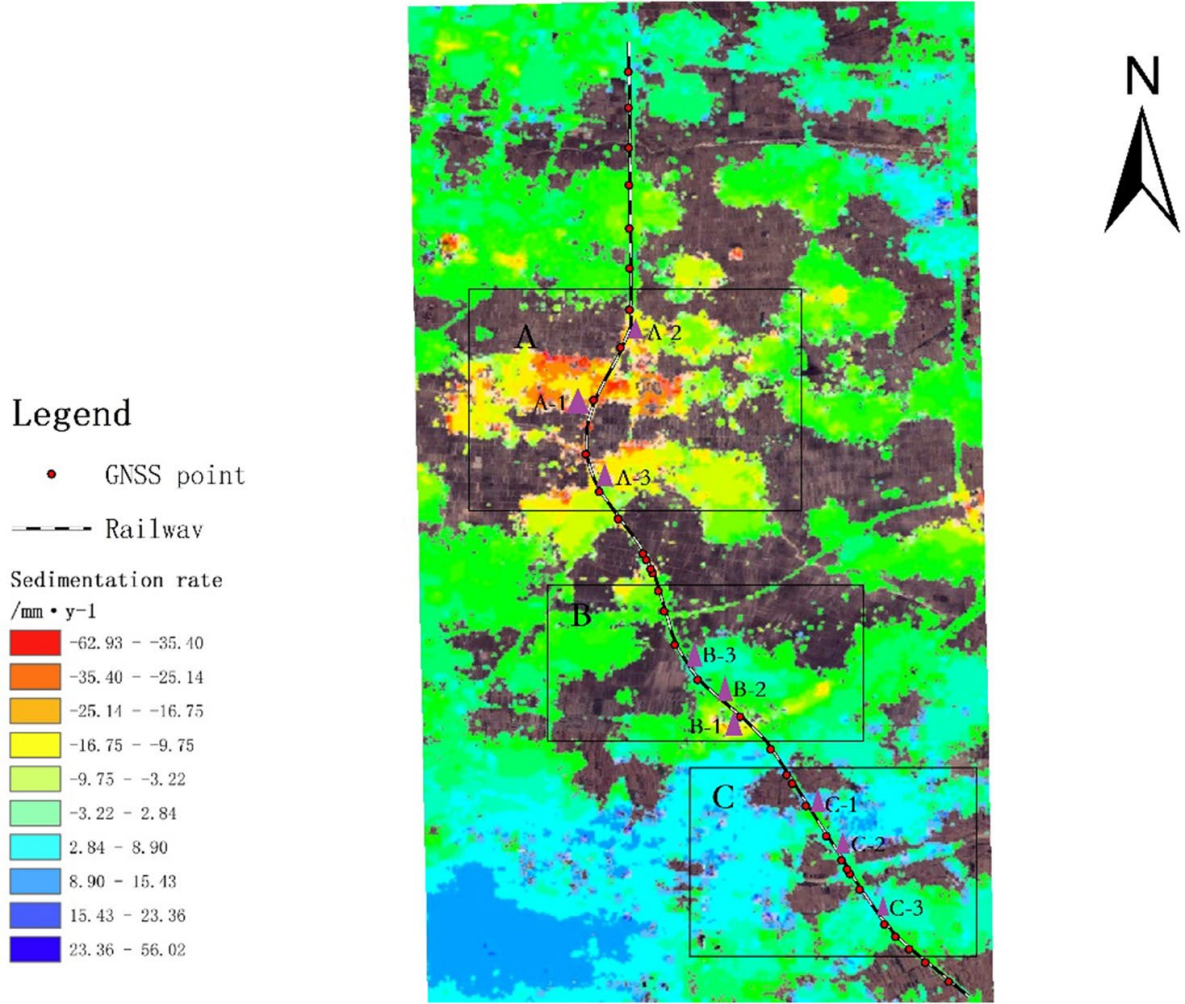
Distribution of GNSS stations along the railway and areas A, B and C.

**Figure 7:**
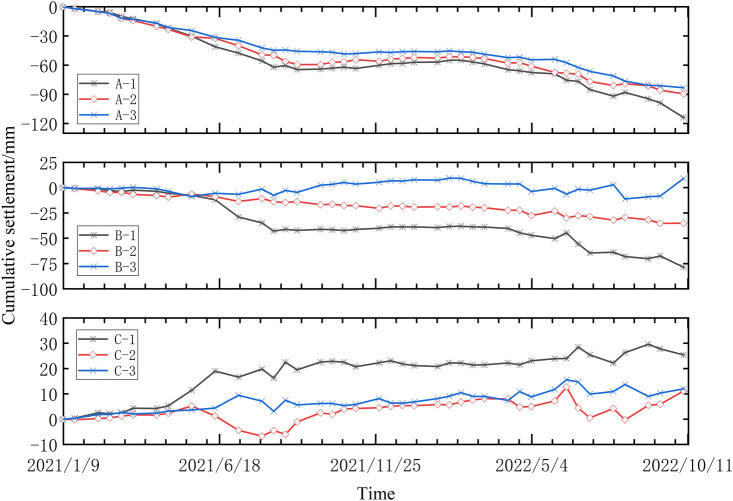
Settlement of feature points in ABC region.

There are three distinct deformations observed along the studied railway area, as depicted in Fig. 6. These subsidence areas are marked as A, B, and C, while three InSAR subsidence feature points are selected in each area and labeled as 1, 2, and 3, respectively. The selection of InSAR settlement feature point 1 in each area is determined using the nearest neighbor method with respect to the GNSS monitoring station near the railway. The time series of settlement measurements for each feature point in every region are presented in Fig. 7. The time series analysis reveals that the settlement trends and cumulative subsidence of the three feature points within area A, located at different positions, are primarily consistent. This suggests a large subsidence range within area A, corroborating the findings depicted in Fig. 5. Notably, characteristic point A-1, situated near the center of the subsidence area and close to the GNSS station, exhibits a maximum cumulative settlement of 110 mm. Feature points A-2 and A-3, situated at the periphery of the subsidence zone, display cumulative subsidence of 90 mm and 80 mm, respectively. During the monitoring period of the three feature points in area A, the cumulative settlement trend demonstrates a sharp increase, followed by a gentler rate, and then another sharp increase. The period of gentler cumulative settlement for the three feature points spans from August 2021 to February 2022, indicating a consistent subsidence trend along the railway lines in area A. The railway section in area B encompasses both uplifted and subsided regions. Among these, feature point B-1 experiences significant cumulative subsidence of 75 mm, while feature point B-2, situated between B-1 and B-3, demonstrates cumulative subsidence of 25 mm. Feature point B-3 represents an uplifted area with a relatively minor cumulative uplift. Area C is predominantly characterized by uplift, albeit at a slower rate compared to the sinking trend observed in area A. The maximum cumulative uplift within this area is observed at feature point C-1, reaching 22 mm, while featuring points C-2 and C-3 exhibit cumulative uplifts of 10 mm each.

**Figure 8:**
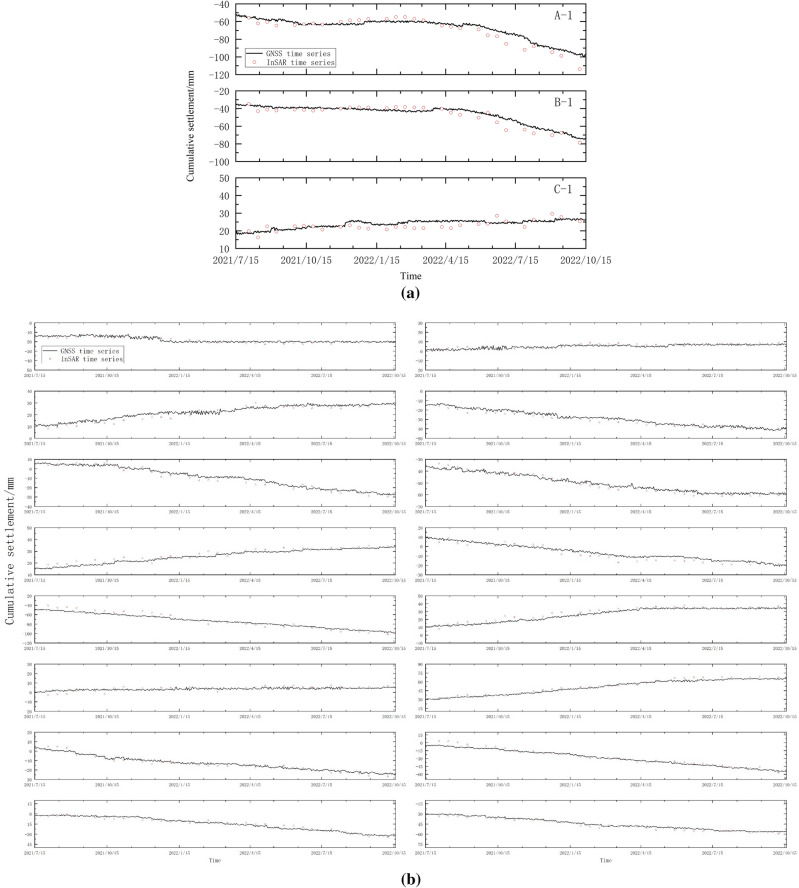
(**a**) Deformation time series of GNSS and InSAR unified coordinate frame of feature points (**b**) Railway along the GNSS and InSAR feature points unified coordinate system deformation time series.

Figure 8a represents the time series data after the coordinate conversion of the GNSS data using Eq. (16). It showcases the unified coordinate frame for the results monitored by the GNSS base stations near feature points A-1, B-1, and C-1, as well as the InSAR results. The results show that the Mean Absolute Error (MAE) of InSAR monitoring settlement data of feature point A-1 is 4.61 mm, at feature point B-1 is 3.83 mm, and at feature point C-1 is 2.28 mm. To enhance the persuasiveness of the results, we selected the remaining GNSS monitoring points and employed the nearest neighbor method to choose the corresponding InSAR points to validate the accuracy of the InSAR results. Due to the constraints of our research scope and our specific focus on areas of interest, there were situations where InSAR results were unavailable near some GNSS stations or the distance to the nearest InSAR feature point exceeded a certain threshold, rendering a direct comparison unfeasible. As a result, we selected the locations and monitoring data of 16 eligible GNSS points and compared them with nearby InSAR feature points using the nearest neighbor method. In Fig. 8b, the time series results of the coordinate transformation are presented for 16 randomly selected GNSS monitoring points along the railway and the adjacent InSAR feature points.

Table 2 shows the MAE and Root Mean Square Error (RMSE) of InSAR points near different GNSS stations. We can find that except for point 3, the MAE and RMSE of the InSAR points are both small. These results affirm the reliability and precision of SBAS-InSAR technology for monitoring settlement data. Therefore, this paper considers the InSAR monitoring data along the railway as the real observation data and conducts retrospective prediction research.

**Table 2:** InSAR accuracy evaluation near GNSS points at different locations.

Points	MAE/mm	RMSE/mm	Points	MAE/mm	RMSE/mm
1	2.15	2.54	9	4.85	5.65
2	1.54	1.74	10	2.90	3.27
3	16.36	16.87	11	1.95	2.31
4	2.46	2.78	12	2.53	2.89
5	2.28	2.56	13	2.12	2.39
6	2.23	2.57	14	3.16	4.23
7	2.19	2.46	15	2.33	2.75
8	3.12	3.43	16	2.15	2.54

## Experiment

### Experimental scheme

In this study, we utilized three prediction models: the ARMA model, the Prophet model, and the EEMD-Prophet prediction method, to predict and compare the InSAR monitoring points along the high-speed railway and its surrounding areas.

Firstly, we utilized GNSS monitoring points along the railway in this area and applied the nearest neighbor method to select the corresponding InSAR settlement point. Subsequently, we conducted a comparative analysis of the settlement trend and the accuracy of InSAR monitoring.

Secondly, we utilized the three prediction models to forecast and compare the feature points along the railway. Initially, we predicted and analyzed the feature points located close to the selected GNSS monitoring points from the previous step and compared the results with the corresponding GNSS monitoring data. Subsequently, for the feature points situated at a greater distance from the GNSS monitoring station, we employed a retrospective prediction method for comparative analysis.

Thirdly, at a regional scale, we selected all InSAR points near Taigu East Railway Station along the railway line. Using the retrospective prediction method, we employed the three prediction methods to generate land subsidence predictions and created a subsidence map. We then compared this map with the original InSAR land subsidence map.

The BeiDou Navigation Satellite System (BDS) measurement and monitoring points deployed along the railway line are utilized to evaluate the accuracy of surface subsidence monitoring using through InSAR technology. BDS monitoring station can measure both horizontal and vertical deformations. The displacement of the BDS base station can be converted into the Line-of-Sight (LOS) direction of InSAR^23^. This conversion is represented by Eq. (15):

15 $${\text{d}}_{{{\text{LOS}}}} = {\text{d}}_{{\text{N}}} \sin \left( {{\uptheta }_{{{\text{inc}}}} } \right)\sin \left( {{\upalpha }_{{\text{h}}} } \right) - {\text{d}}_{{\text{E}}} \sin \left( {{\uptheta }_{{{\text{inc}}}} } \right)\cos \left( {{\upalpha }_{{\text{h}}} } \right) + {\text{d}}_{{\text{U}}} \cos \left( {{\uptheta }_{{{\text{inc}}}} } \right)$$

In Eq. (15), ${\text{d}}_{{{\text{LOS}}}}$ represents the satellite line of sight direction, ${\upalpha }_{{\text{h}}}$ is the satellite radar heading angle, ${\uptheta }_{{{\text{inc}}}}$ is the satellite radar incident angle, ${\text{d}}_{{\text{N}}}$, ${\text{d}}_{{\text{E}}}$, and ${\text{d}}_{{\text{U}}}$ are the north, east, and displacement in the upward direction. Assuming that the horizontal displacement of the BDS station is negligible^24^, it can be simplified as follows:

16 $${\text{d}}_{{{\text{LOS}}}} = {\text{d}}_{{\text{U}}} \cos \left( {{\uptheta }_{{{\text{inc}}}} } \right)$$

To assess the prediction accuracy of feature points, we employ RMSE and MAE as evaluation metrics. The two parameters are defined as follows:

17 $${\text{RMSE}} = \sqrt {\frac{{\mathop \sum \nolimits_{{{\text{i}} = 1}}^{{\text{N}}} \left( {\hat{Z} - {\text{Z}}} \right)^{2} }}{{\text{N}}}}$$

18 $${\text{MAE}} = \frac{{\mathop \sum \nolimits_{i = 1}^{N} \left| {\left( {\hat{Z} - {\text{Z}}} \right)} \right|}}{N}$$

where ${\hat{\text{Z}}}$ is the predicted value, Z is the measured value. ${\text{RMSE}} \in \left( {0, + \infty } \right]$, the closer the matter is to 0, the more consistent the expected value is with the measured value. ${\text{MAE}} \in \left( {0, + \infty } \right]$, the closer the value is Closer to 0, then it reflects the combination model is better.

For intra-regional prediction, ${\text{RMSE}}_{{{\text{zone}}}}$ and coefficient of determination ${\text{R}}^{2}$^25^ are used to evaluate the overall prediction accuracy of the model. The two parameters are defined as follows:

19 $${\text{RMSE}}_{{{\text{zone}}}} = \sqrt {\frac{{\mathop \sum \nolimits_{{{\text{i}} = 1}}^{{\text{N}}} \left( {{\text{Z}}_{{\text{t}}} \left( {{\text{x}}_{{\text{i}}} ,{\text{y}}_{{\text{i}}} } \right) - {\text{Z}}_{{{\text{I}},{\text{t}}}} \left( {{\text{x}}_{{\text{i}}} ,{\text{y}}_{{\text{i}}} } \right)} \right)^{2} }}{{\text{N}}}}$$

20 $${\text{R}}^{2} \left( {{\text{Z}}_{{\text{t}}} ,\hat{Z}_{{{\text{I}},{\text{t}}}} } \right) = 1 - \frac{{\mathop \sum \nolimits_{{{\text{i}} = 0}}^{{\text{N}}} \left( {{\text{Z}}_{{\text{t}}} \left( {{\text{x}}_{{\text{i}}} ,{\text{y}}_{{\text{i}}} } \right) - \hat{Z}_{{{\text{I}},{\text{t}}}} \left( {{\text{x}}_{{\text{i}}} ,{\text{y}}_{{\text{i}}} } \right)} \right)^{2} }}{{\mathop \sum \nolimits_{{{\text{i}} = 0}}^{{\text{N}}} \left( {{\text{Z}}_{{\text{t}}} \left( {{\text{x}}_{{\text{i}}} ,{\text{y}}_{{\text{i}}} } \right) - \overline{Z}_{{{\text{I}},{\text{t}}}} \left( {{\text{x}}_{{\text{i}}} ,{\text{y}}_{{\text{i}}} } \right)} \right)^{2} }}$$

Among them, ${\text{Z}}_{{\text{t}}} \left( {{\text{x}}_{{\text{i}}} ,{\text{y}}_{{\text{i}}} } \right)$ represents the predicted settlement value at time t and position $\left( {{\text{x}}_{{\text{i}}} ,{\text{y}}_{{\text{i}}} } \right)$, while ${\text{Z}}_{{{\text{I}},{\text{t}}}} \left( {{\text{x}}_{{\text{i}}},{\text{y}}_{{\text{i}}} } \right)$ represents the observed settlement value obtained from InSAR monitoring at time t and position $\left( {{\text{x}}_{{\text{i}}} ,{\text{y}}_{{\text{i}}} } \right)$. The coefficient of determination, ${\text{R}}^{2}$, quantifies the degree to which the independent variable can explain the changes in the dependent variable. A value closer to 1 indicates a stronger fit of the predictive model. $\hat{Z}_{{{\text{I}},{\text{t}}}} \left( {{\text{x}}_{{\text{i}}} ,{\text{y}}_{{\text{i}}} } \right)$ represents the true value of the observed data, this article refers to the InSAR observed value. $\overline{Z}_{{{\text{I}},{\text{t}}}} \left( {{\text{x}}_{{\text{i}}} ,{\text{y}}_{{\text{i}}} } \right)$ is the mean of the observed data. ${\text{Z}}_{{\text{t}}}$ is the predicted value of the model.

### Single point forecasting and analysis

The subsidence time series data of feature points obtained by SBAS-InSAR technology are used as actual values for retrospective prediction research. Among them, the monitoring data from the initial 35 periods were selected as the sample data to predict the settlement for the next five periods, and the actual monitoring results were compared with the predicted results.

**Figure 9:**
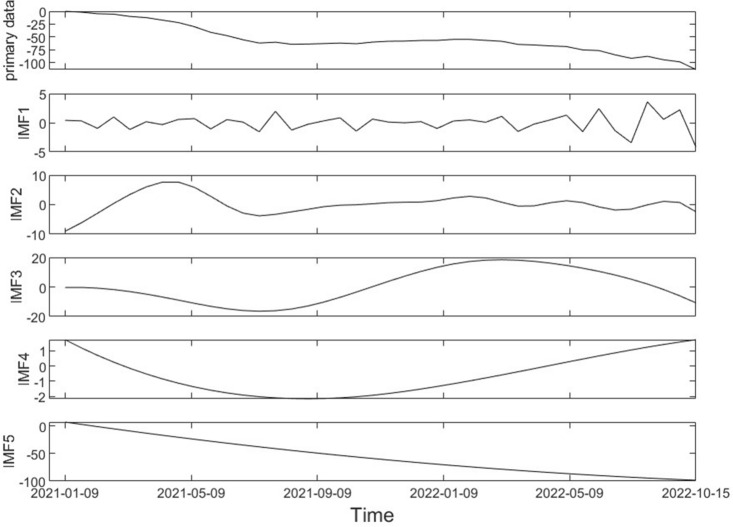
EEMD decomposition InSAR timing.

Firstly, EEMD decomposition is performed on the time series data of SBAS-InSAR monitoring points, resulting in the extraction of multiple IMF components and a residual component from the time series signal. This process allows us to capture the local characteristics of the monitoring points at different times, enabling the interpolation and prediction of their time series. By subjecting InSAR time series data to EEMD, the original signal can be approximated, and its components, referred to as IMF, can be extracted. Figure 9 presents a schematic diagram of the EEMD-decomposed IMF for a specific timing monitoring point of SBAS-InSAR. The time series acquired through SBAS-InSAR technology is decomposed into five components, with each IMF component reflecting distinct information and containing oscillation characteristics of the original sequence. IMF1 represents the component with the highest frequency, while the frequency decreases sequentially for the subsequent IMF components. IMF5, being the component with the lowest frequency, captures the trend information of the original sequence. The downward trend observed in IMF5 aligns with the overall trend of the original sequence.

Table 1 clearly shows the SAR satellite has encountered data loss during a specific time interval, resulting in gaps in the subsidence time series used for SBAS-InSAR analysis of surface subsidence. These missing images present significant challenges to accurately assessing subsidence. In this study, the selected subsidence period spans from August 8, 2022, to October 7, 2022. Leveraging the periodicity of Sentinel-1A remote sensing images acquired every 12 days, we can infer the missing image timestamps by referencing to the information presented in Table 1. Specifically, the temporal gaps correspond to August 20, 2022, and September 25, 2022. Based on this premise, the initial selection for verification of the EEMD-Prophet prediction method includes feature points A-1, B-1, and C-1, which are in proximity to the GNSS base station. These selected feature points serve as the basis for conducting prediction experiments using the proposed EEMD-Prophet method. The predicted values for the missing time intervals are then compared with the corresponding GNSS measurements during those periods.

**Figure 10:**
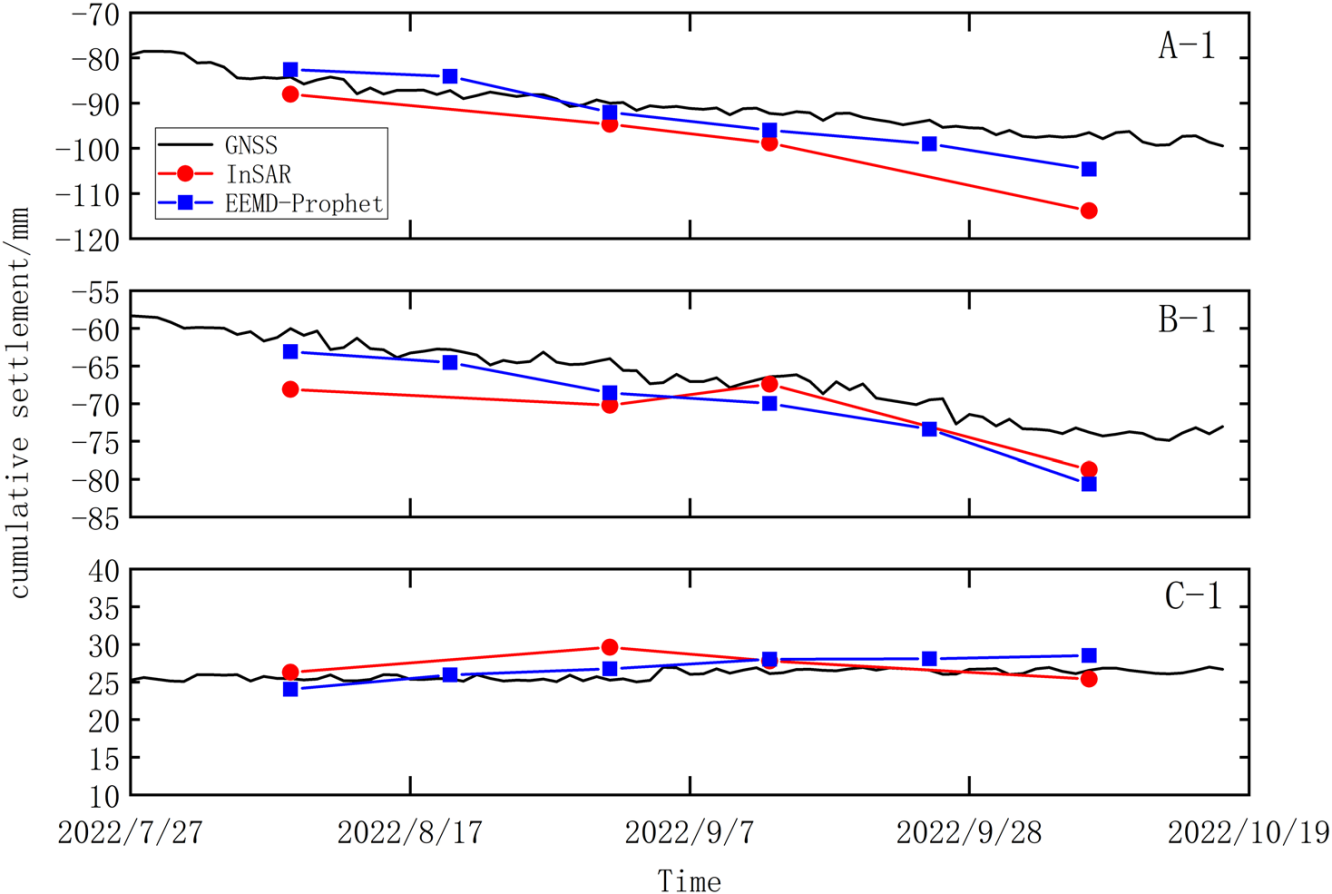
Comparison of EEMD-Prophet prediction results with GNSS.

Figure 10 showcases the settlement prediction of feature points A-1, B-1, and C-1 utilizing the EEMD-Prophet method. It is evident that the projected values generated by the EEMD-Prophet method closely align with the GNSS measured values during the initial three prediction periods. However, the accuracy of the predictions gradually diminishes as the forecasting horizon extends. By incorporating span interpolation for the prediction time, the EEMD-Prophet method effectively anticipates the missing image data of SBAS-InSAR. The forecast results and corresponding relative error statistics for feature points A-1, B-1, and C-1 through the implementation of the EEMD-Prophet method are presented in Table 3. Notably, the missing images in InSAR correspond to August 20, 2022, and September 25, 2022.

**Table 3:** Predictive value and relative error of EEMD-Prophet combined model.

Time	Point A-1		Point B-1		Point C-1	
	Predictive value/mm	Relative error/%	Predictive value/mm	Relative error/%	Predictive value/mm	Relative error/%
2022/8/8	− 82.57	1.97	− 63.11	5.12	24.02	5.63
2022/8/20	− 84.06	3.61	− 64.53	2.73	25.95	2.06
2022/9/1	− 92.03	2.25	− 68.57	7.12	26.76	5.96
2022/9/13	− 96.00	4.01	− 69.97	5.34	28.01	7.29
2022/9/25	− 99.04	5.62	− 73.41	5.65	28.09	5.74
2022/10/7	− 104.60	8.37	− 80.62	9.22	29.50	7.34
Average value		4.31		5.86		5.67

To validate the effectiveness of the proposed method for settlement prediction along the railway, a single prediction method using EEMD-Prophet is insufficient. In this study, we conducted prediction experiments using the Prophet model, the ARMA model^5,6^, and the EEMD-Prophet model, as proposed in this paper. The experimental results were compared and analyzed to assess the feasibility of the proposed method.

For the purpose of comparative analysis, we selected feature points A-2, A-3, B-2, B-3, C-2, and C-3. These points were chosen because they are situated at a significant distance from the GNSS base station, and the accuracy of SBAS-InSAR technology meets the required standards. Consequently, the InSAR monitoring values of these feature points were employed as the actual observation data, and a retrospective prediction approach was adopted for comparative analysis and verification. The last five periods were designated as the forecast timeframe and compared against the actual observed values.

**Figure 11:**
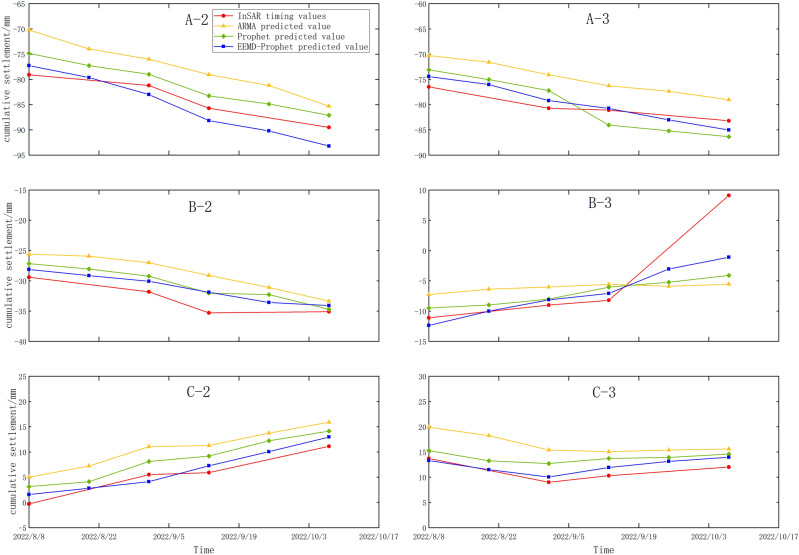
Comparison of prediction models.

The prediction results depicted in Fig. 11 illustrate that the EEMD-Prophet prediction values exhibit closer proximity to the actual monitoring values compared to the Prophet model and the ARMA model. Overall, the EEMD-Prophet method demonstrates superior prediction performance. It achieves the highest accuracy during the initial three prediction intervals, gradually decreasing accuracy as the prediction time extends.

To analyze the effectiveness of the EEMD-Prophet model prediction method, various indicators such as RMSE and MAE were employed to compare and analyze the prediction outcomes. As shown in Table 4, the EEMD-Prophet prediction method outperforms the ARMA model and the Prophet model in terms of single-point prediction accuracy. The average RMSE and MAE values of the EEMD-Prophet method are recorded as 2.41 mm and 1.47 mm, respectively. The RMSE improves by 58.01% and 32.3% compared to the ARMA model and the Prophet model, respectively. Similarly, the MAE exhibits improvements of 62.69% and 33.78% when compared to the ARMA model and the Prophet model, respectively.

**Table 4:** Prediction accuracy of different models.

Modle point	ARMA		Prophet		EEMD-Prophet	
	RMSE/mm	MAE/mm	RMSE/mm	MAE/mm	RMSE/mm	MAE/mm
A-2	6.48	6.23	2.95	2.82	2.57	2.45
A-3	5.54	3.64	3.25	2.16	1.58	0.96
B-2	4.44	2.76	2.38	1.42	2.09	1.24
B-3	7.84	4.02	6.76	2.99	5.18	2.24
C-2	4.80	3.50	3.11	2.05	1.64	1.08
C-3	5.34	3.48	2.92	1.87	1.38	0.83
average value	5.74	3.94	3.56	2.22	2.41	1.47

### Regional forecast and analysis

**Figure 12:**
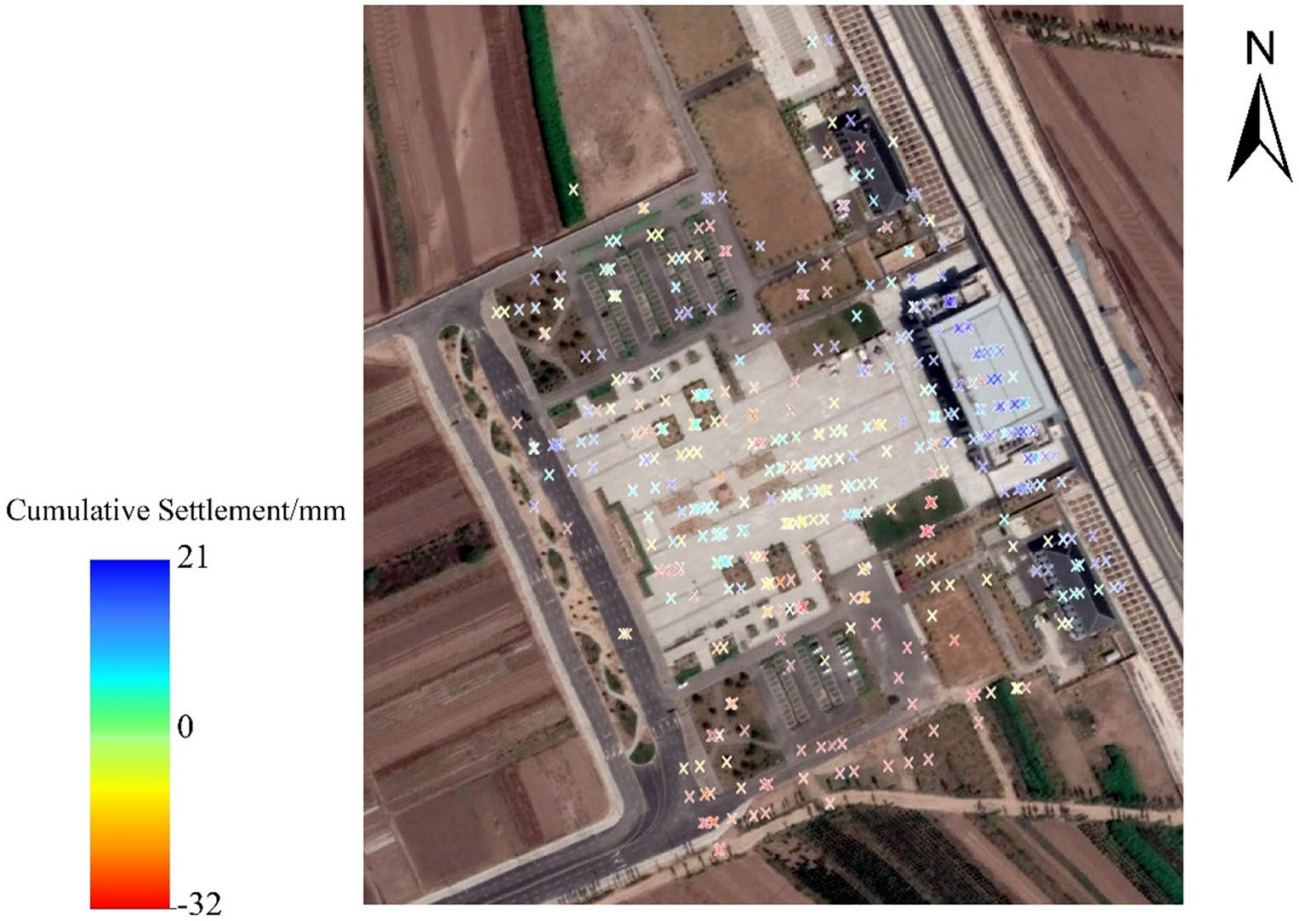
Distribution of InSAR monitoring points at Taigu East Station.

**Figure 13:**
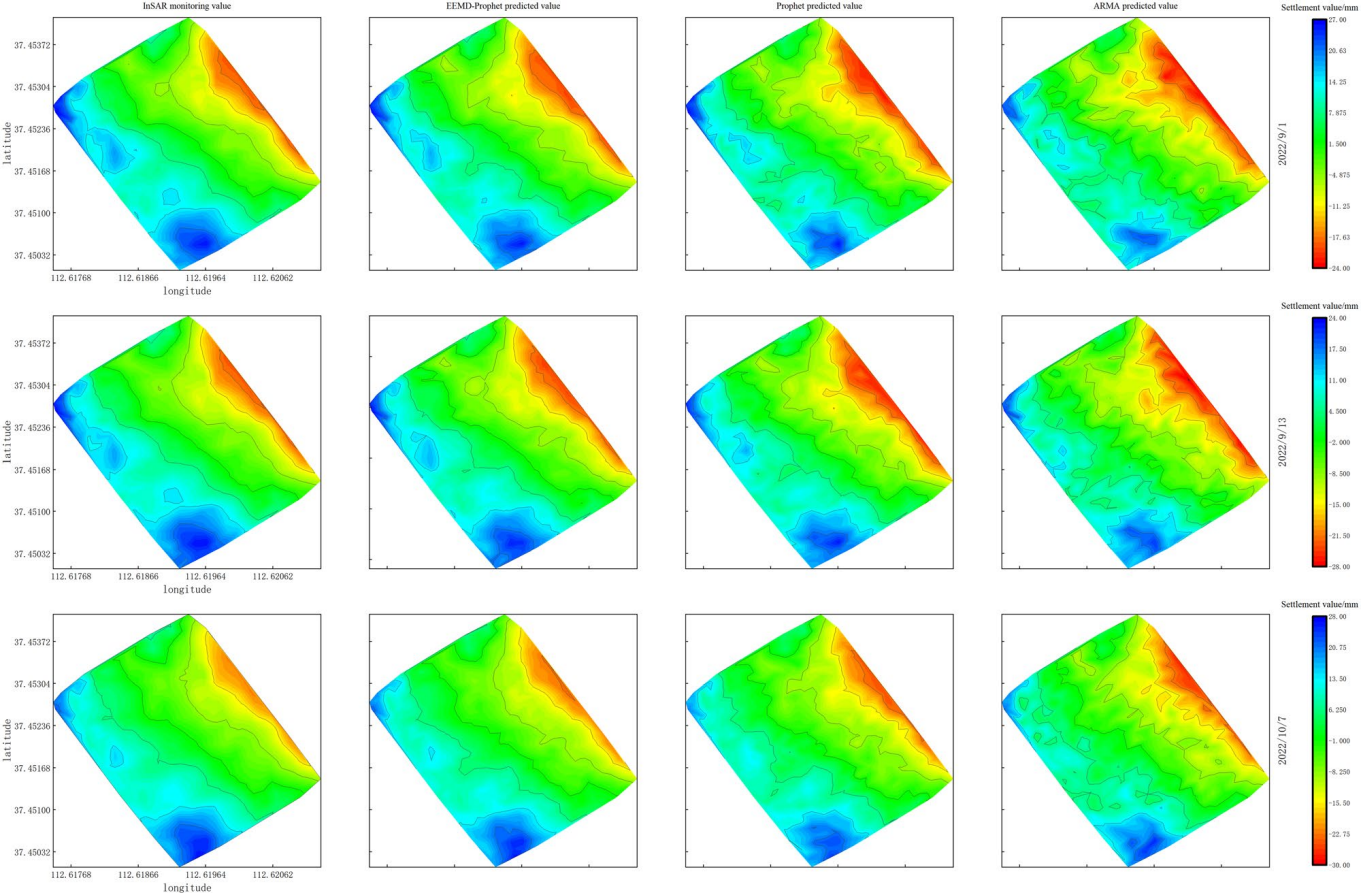
Land subsidence of Taigu East Station regional prediction model.

In Sect. 4.2, we employed the EEMD-Prophet, Prophet, and ARMA models to predict the settlement time series at a single feature point. In this section, we focus on the prediction of settlement at Taigu East Station, a significant area along the Taijiao high-speed railway. The geographical location of Taigu East Station is depicted in Fig. 4 Furthermore, Fig. 12 displays the region encompassing Taigu East Station and its 455 corresponding InSAR monitoring points in Google Earth Pro (V7.3.6.9345; https://www.google.com/earth/about/versions/#download-pro). These monitoring points are utilized for prediction and evaluation using the EEMD-Prophet, Prophet, and ARMA models. Figure 13 is a visual representation of ground settlement generated through Kriging interpolation^26^ after predicting the monitoring points in the Taigu East Station area.

Upon examination, two prominent uplift areas and one notable subsidence area are identified within the vicinity of Taigu East Station. The subsidence area is situated at the intersection of the railway line and Taigu East Station. This observation suggests that, once trains commence operations after railway construction is completed, they exert pressure on the foundation, resulting in ground subsidence. Of particular note, the proximity of the railway station to the railway line indicates that the train load on this site may be substantial, resulting in noticeable settlement.

Figure 13 presents the settlement prediction of the Taigu East Station area using different prediction models, resulting in a ground settlement map. The EEMD-Prophet prediction model exhibits consistency with the actual InSAR land subsidence. However, as the prediction time increases, the established subsidence map gradually deviates from the actual InSAR ground subsidence map. To assess the accuracy and precision of each prediction method for regional prediction, we utilize the ${{\text{RMSE}}}_{{\text{zone}}}$, coefficient of determination ${{\text{R}}}^{2}$, and the time required for Taigu East Station prediction. Table 5 displays the average indicators of the prediction models at three prediction time points.

**Table 5:** Evaluation indicators of the model in regional prediction.

Evaluation index	${{\text{RMSE}}}_{{\text{zone}}}$	${R}^{2}$	Time
EEMD-Prophet	3.23mm	0.79	7min28s
Prophet	3.69mm	0.74	7min06s
ARMA	5.72mm	0.68	8min32s

When examining the results, it becomes evident that the EEMD-Prophet prediction model outperforms both the ARMA model and the Prophet model in terms of regional prediction accuracy, achieving an ${{\text{RMSE}}}_{{\text{zone}}}$ of 3.23 mm. Compared to the ARMA model and the Prophet model, the EEMD-Prophet model yields a improve in ${{\text{RMSE}}}_{{\text{zone}}}$ by 43.53% and 12.47%, respectively. Moreover, the coefficient of determination for the EEMD-Prophet model is closer to 1, reaching 0.79, surpassing the other two prediction models. While the EEMD-Prophet model requires an additional 22s for EEMD decomposition on top of the Prophet model, its running speed exhibits a 12.45% improvement compared to the calculation time of the ARMA model.

## Conclusions

This paper utilizes 40 SAR images to monitor surface subsidence in the study area through the SBAS method. By analyzing the 40-period time-series subsidence changes and cumulative subsidence, the SBAS monitoring values are employed as the time-series data sample for retrospective forecasting research. For forecasting research, the EEMD-Prophet forecasting model, the Prophet model, and the ARMA model are employed. Overall, the combined model demonstrates higher prediction accuracy compared to the Prophet model and the ARMA model.

In the study of SBAS-InSAR settlement, this paper introduces the EEMD-Prophet model, which combines EEMD decomposition and Prophet prediction, to interpolate and predict InSAR monitoring time series. The proposed method exhibits strong adaptability and provides favorable prediction results for analyzing similar subsidence time series signal data. However, it should be noted that the decomposition and prediction process of the EEMD-Prophet model introduces an additional workload. Throughout the research process, it is crucial to consider that settlement changes along the high-speed railway are influenced by a variety of factors. Therefore, there is a need for further investigation into the development of an efficient and self-adaptive prediction method capable of capturing sudden changes over time.

In addition to the methods explored in this study, it's worth noting that machine learning techniques, such as LSTM, have shown promise in the prediction of ground subsidence. LSTM, with its ability to capture complex temporal dependencies in data, can be particularly useful in modeling and predicting land subsidence time series, especially in urban areas and regions influenced by anthropic activities. While the EEMD-Prophet model demonstrated enhanced predictive accuracy, combining it with machine learning methods could potentially offer even more robust results, especially in scenarios where sudden changes or non-linear patterns in subsidence need to be captured in a timely manner. This integration of techniques could represent an exciting avenue for further research in the field of land subsidence analysis and prediction.

## Data Availability

The datasets used and/or analyzed during the current study are available from the corresponding author on reasonable request.
